# Coffee Consumption and Risk of Colorectal Cancer: The Japan Collaborative Cohort Study

**DOI:** 10.2188/jea.JE20130168

**Published:** 2014-09-05

**Authors:** Hiroya Yamada, Miyuki Kawado, Norihiro Aoyama, Shuji Hashimoto, Koji Suzuki, Kenji Wakai, Sadao Suzuki, Yoshiyuki Watanabe, Akiko Tamakoshi, the JACC Study Group

**Affiliations:** 1 Department of Hygiene, Fujita Health University School of Medicine, Toyoake, Aichi, Japan; 1 藤田保健衛生大学医学部衛生学講座; 2 Department of Public Health, Fujita Health University School of Health Sciences, Toyoake, Aichi, Japan; 2 藤田保健衛生大学医療科学部公衆衛生学; 3 Department of Preventive Medicine, Nagoya University Graduate School of Medicine, Nagoya, Japan; 3 名古屋大学大学院医学研究科予防医学; 4 Department of Public Health, Nagoya City University Graduate School of Medical Sciences, Nagoya, Japan; 4 名古屋市立大学大学院医学研究科公衆衛生学分野; 5 Department of Epidemiology for Community Health and Medicine, Kyoto Prefectural University of Medicine Graduate School of Medical Science, Kyoto, Japan; 5 京都府立医科大学大学院医学研究科地域保健医療疫学; 6 Department of Public Health, Hokkaido University Graduate School of Medicine, Sapporo, Japan; 6 北海道大学大学院医学研究科公衆衛生学分野

**Keywords:** coffee, colorectal cancer, incidence, prospective study, the Japan Collaborative Cohort Study

## Abstract

**Background:**

Epidemiologic studies have reported coffee consumption to be associated with various health conditions. The purpose of this study was to examine the relationship of coffee consumption with colorectal cancer incidence in a large-scale prospective cohort study in Japan.

**Methods:**

We used data from the Japan Collaborative Cohort Study for Evaluation of Cancer Risk (JACC Study). Here, we analyzed a total of 58 221 persons (23 607 men, 34 614 women) followed from 1988 to the end of 2009. During 738 669 person-years of follow-up for the analysis of colorectal cancer risk with coffee consumption at baseline, we identified 687 cases of colon cancer (355 males and 332 females) and 314 cases of rectal cancer (202 males and 112 females). We used the Cox proportional-hazard regression model to estimate hazard ratio (HR).

**Results:**

Compared to those who consumed less than 1 cup of coffee per day, men who consumed 2–3 cups of coffee per day had an HR of 1.26 (95% confidence interval [CI] 0.93–1.70), and men who consumed more than 4 cups of coffee per day had an HR of 1.79 (95% CI 1.01–3.18). A statistically significant increase in the risk of colon cancer was associated with increasing coffee consumption among men (*P* for trend = 0.03). On the other hand, coffee consumption in women was not associated with incident risk of colon cancer. Coffee consumption was also not associated with rectal cancer incidence in men or women.

**Conclusions:**

This large-scale population-based cohort study showed that coffee consumption increases the risk of colon cancer among Japanese men.

## Abstract (ja)

**【背景】:**

疫学研究によりコーヒー摂取と様々な健康状態との関連が報告されている。今回、コーヒー摂取と大腸がんとの関連を前向きコホート研究により評価することを目的とする。

**【方法】:**

JACC Studyにおける1988年から2009年まで追跡した58,221人（男性23,607人、女性34,614人）のデータを用いた。ベースライン後の738,669人年の追跡により、結腸がん罹患687人（男性355人、女性332人）、直腸がん314人（男性男性202人、女性112人）が観察されている。大腸がん罹患の有無に基づいて、ハザード比と95％信頼区間を、コックス回帰により交絡変数を調整して推定した。

**【結果】:**

結腸がんについて、コーヒー摂取が1日1杯以下の男性に比べて、1日2～3杯のコーヒー摂取をする男性でHRが1.26 (95%信頼区間0.93-1.70)、1日4杯以上の男性でHR が1.79 (95%信頼区間 1.01-3.18)を示した。一方、女性では有意な関連は認められなかった。また、男女とも直腸がんでは関連が認められなかった。

**【結論】:**

JACC studyの解析結果では、男性においてコーヒー摂取は結腸がんリスクの上昇を示した。

## INTRODUCTION

Colorectal cancer is already one of the most common cancers in Western countries and is rapidly increasing in incidence across Asia.^1^ This increase is considered to be associated with changes in environmental factors such as dietary habits and lifestyle. Therefore, primary prevention of colorectal cancer worldwide is a considerable public health concern.^2^

Coffee is one of the most widely consumed beverages in the world. Recent national data from Japan have revealed that the average per capita coffee consumption is about 127.1 g per day (Japan Ministry of Health Labour and Welfare, 2010). Therefore, even small effects of coffee on individuals could have a large effect on general public health. A number of epidemiologic studies have investigated the relationship between coffee consumption and colorectal cancer, but findings regarding the effect of coffee on the incidence of this cancer have been inconsistent.^3^^–^^13^ A meta-analysis by Je et al reported no association between coffee consumption and colon cancer risk, whereas another meta-analysis by Li et al reported a significantly increased risk.^14^^,^^15^ Further, most previous studies have been conducted in Western countries.^3^^–^^10^ The three major cohort studies from Japan on the relationship between coffee consumption and the risk of colorectal cancer^11^^–^^13^ have also reported inconsistent results: the Japan Public Health Center (JPHC) Cohort Study and Takayama Cohort Study suggested that coffee consumption may lower the risk of colon cancer in women,^11^ whereas the Miyagi Cohort Study concluded that consumption was not associated with risk of colorectal cancer in either sex.^12^^,^^13^

The Japan Collaborative Cohort Study for Evaluation of Cancer Risk (JACC Study) is a large-scale population-based cohort study. The study has provided many findings on cancer risk associated with lifestyle and living conditions in the Japanese population but has not reported results on the association between coffee consumption and colorectal cancer incidence.^16^

The aim of this study was to examine the relationship of coffee consumption with colon and rectal cancer incidence in a large-scale prospective cohort study in Japan.

## METHODS

### Study subjects and data collection

Details of the study concept and design of the JACC Study have been described elsewhere.^16^ Briefly, the JACC Study was started between 1988 and 1990 and enrolled subjects living in 45 areas in Japan. A total of 110 585 Japanese subjects (46 395 men and 64 190 women) aged 40–79 at baseline were followed to the end of 2009.

We analyzed colorectal cancer risk with coffee consumption at baseline using data from a baseline survey. Of 110 585 participants at baseline, subjects for the present analysis were restricted to 65 042 participants who lived in the 24 areas in which information on cancer incidence was available. We excluded subjects in the study whose baseline questionnaire did not include a section on coffee consumption, who skipped questions about coffee consumption, or who had a history of colorectal cancer. After exclusion, 58 221 subjects (23 607 men, 34 614 women) remained for the final analysis.

Information about coffee consumption and other lifestyle factors was obtained using a self-administered questionnaire. Subjects were grouped into four categories according to daily coffee intake at baseline, namely: less than 1 cup, 1 cup, 2–3 cups, or 4 or more cups a day. These four categories were determined by reference to a previous study.^17^ The question regarding coffee consumption was previously assessed by a validation study, which reported a strong agreement with 12-day weighted dietary records (Spearman correlation: 0.81).^18^

### Follow-up

Subjects were followed from the baseline survey until 2009. Individuals who moved away from the study area were treated as study dropouts, because deaths after such moves could not be confirmed in our follow-up system. The occurrence of cancer was confirmed from population-based cancer registries or by reviewing the records of local major hospitals. We defined colon cancer as C18 and rectal cancer as C20 according to the International Statistical Classification of Diseases and Related Health Problems 10th Revision (http://www.who.int/classifications/icd/en/). The study protocol was approved by the Ethical Board of Nagoya University School of Medicine.

### Analysis

For the analysis of the association between colorectal cancer risk and coffee consumption, hazard ratios (HRs) and 95% confidence intervals (CIs) were calculated using Cox’s proportional hazards model adjusted for 5-year age groups by gender. In multivariate analyses, we further adjusted for several factors known to be associated with colorectal cancer and/or coffee consumption, including smoking status (current smoker of more than 20 cigarettes/day, current smoker of at least 1 cigarette/day but no more than 20 cigarettes/day, former smoker, or never smoker), daily walking duration (walking more than 30 min per day or not), education (attended school up to 15–18 years old, or >18 years old), body mass index (BMI; <18.5 kg/m^2^, 18.5 kg/m^2^ to 25 kg/m^2^, or >25.0 kg/m^2^), alcohol intake (daily drinker, less than daily drinker, former drinker, nondrinker), family cancer history (yes or no), and meat consumption (high consumption of beef and pork, middle consumption, or low consumption). Data for the above factors were self-reported. For all covariates, missing values were treated as an additional category of variable and were included in the model. The linear trend in incident risk was assessed by treating the number of cups of coffee intake per day as an ordinary variable. All analyses were performed using the SAS statistical package, version 9.3 (SAS Institute, Cary, NC, USA).

In addition, we performed stratified analyses by smoking status (never smoker or current smoker), BMI (<18.5 kg/m, 18.5 kg/m^2^ to 25 kg/m^2^, or >25.0 kg/m^2^), meat consumption (high consumption of beef and pork, first tertile; middle consumption, second tertile; and low consumption, third tertile), age (40–59 years or 60–69 years), and drinking status (drinker or never drinker).

## RESULTS

Baseline characteristics of the study cohort by coffee consumption are presented in Table 1. Subjects with high coffee consumption were younger, better educated, and more likely to be smokers, alcohol drinkers, and to regularly eat beef or pork. Similar trends were obtained for men and women. However, unlike in men, alcohol drinking in women increased with an increase in coffee consumption, albeit the proportion of drinkers was relatively low.

**Table 1.** Characteristics of subjects for analysis of coffee consumption

Characteristics at baseline survey		Men					Women				
	
		<1 cup/day	1 cup/day	2–3 cups/day	≥4 cups/day	*P*	<1 cup/day	1 cup/day	2–3 cups/day	≥4 cups/day	*P*
Number of subjects		15 569	3172	4116	750		23 345	5844	4932	493	

Age, years; mean (standard deviation)		59.0 (10.0)	57.3 (10.4)	54.0 (9.9)	51.4 (9.6)	<0.0001	60.0 (9.4)	57.2 (10.0)	53.8 (9.5)	49.9 (8.5)	<0.0001

Smoking habit	Current (%)	47.7	51.6	65.8	80.9	<0.0001	3.5	5.4	10.3	32.5	<0.0001
	Former (%)	28.9	28.4	20.1	12.9		1.3	1.8	2.2	2.7	
	Never (%)	23.3	20.1	14.0	6.3		95.2	92.8	87.5	64.9	

Drinking habit	Current (%)	76.1	76.5	73.8	62.6	<0.0001	19.7	30.0	35.0	38.0	<0.0001
	Former (%)	6.7	5.5	5.1	6.9		1.7	1.9	2.0	4.8	
	Never (%)	17.2	18.0	21.1	30.5		78.6	68.1	63.0	57.3	

Education:	High (%)	14.9	19.9	21.1	26.1	<0.0001	8.4	10.3	12.3	15.4	<0.0001

Family history of colorectal cancer:	Yes (%)	2.1	2.5	2.3	2.7	0.27	2.6	2.7	2.3	2.0	0.46

Body mass index:	<18.5 kg/m^2^ (%)	5.6	4.9	4.8	6.4	0.24	6.7	5.8	5.4	7.6	<0.0001
	≥25 kg/m^2^ (%)	18.3	18.0	18.6	19.4		23.1	20.4	20.7	15.2	

Walking time:	≥30 min/day (%)	58.9	64.7	64.9	64.8	<0.0001	59.7	68.0	67.7	66.9	<0.0001

Regular consumption of meat:	Low (%)	39.3	35.1	33.0	29.6	<0.0001	39.3	32.6	29.2	32.1	<0.0001
	Middle (%)	35.0	38.5	42.8	41.0		34.3	41.3	41.0	31.2	
	High (%)	25.7	26.4	24.2	29.5		26.4	26.1	29.7	36.8	

During 738 669 person-years of follow-up, we identified 687 cases of colon cancer (355 males and 332 females) and 314 cases of rectal cancer (202 males and 112 females). Table 2 shows the HRs and 95% CIs for colorectal cancer incidence by coffee consumption. A statistically significant increase in the risk of colon cancer with increasing levels of coffee consumption was seen among men (*P* for trend = 0.03). Compared with men who consumed less than 1 cup of coffee per day, men who consumed 2–3 cups per day showed an unadjusted HR of 1.26 (95% CI 0.94–1.69) and an HR of 1.26 (95% CI 0.93–1.70) after multivariable adjustment for potential confounding factors, and men who consumed more than 4 cups of coffee per day showed an unadjusted HR of 1.72 (95% CI 0.99–3.02) and an HR of 1.79 (95% CI 1.01–3.18) after multivariable adjustment for potential confounding factors. In contrast, coffee consumption was not associated with incident risk of colorectal cancer in women. Compared with women who consumed less than 1 cup of coffee per day, women who consumed 2–3 cups of coffee per day showed an unadjusted HR of 0.90 (95% CI 0.60–1.34) and a HR of 0.86 (95% CI 0.57–1.30) after multivariable adjustment for potential confounding factors; women who consumed more than 4 cups of coffee per day showed an unadjusted HR of 2.16 (95% CI 0.88–5.30) and a HR of 2.02 (95% CI 0.81–5.03) after multivariable adjustment for potential confounding factors. Coffee consumption was not associated with increased risk of rectal cancer in men or women.

**Table 2.** Hazard ratio and 95% confidence interval for colorectal cancer incidence for analysis of coffee consumption

Coffee consumption	Men					Women				
	
	Number of cases	Age adjusted HR (95% confidence interval)	*P* value	Multivariable adjusted HR^a^ (95% confidence interval)	*P* value	Number of cases	Age adjusted HR (95% confidence interval)	*P* value	Multivariable adjusted HR^a^ (95% confidence interval)	*P* value
For colon cancer incidence										
<1 cup/day	240	1.00		1.00		254	1.00		1.00	
1 cup/day	44	1.08 (0.79–1.50)	0.63	1.06 (0.76–1.47)	0.73	46	1.03 (0.75–1.42)	0.84	1.00 (0.72–1.37)	0.98
2–3 cups/day	58	1.26 (0.94–1.69)	0.12	1.26 (0.93–1.70)	0.13	27	0.90 (0.60–1.34)	0.59	0.86 (0.57–1.30)	0.49
≥4 cups/day	13	1.72 (0.99–3.02)	0.06	1.79 (1.01–3.18)	0.05	5	2.16 (0.88–5.30)	0.09	2.02 (0.81–5.03)	0.13
*P* for trend			0.02		0.03			0.77		0.96

For rectum cancer incidence										
<1 cup/day	139	1.00		1.00		82	1.00		1.00	
1 cup/day	28	1.19 (0.79–1.78)	0.41	1.19 (0.79–1.80)	0.40	13	0.85 (0.47–1.53)	0.58	0.88 (0.48–1.59)	0.67
2–3 cups/day	30	1.08 (0.72–1.62)	0.71	1.12 (0.75–1.70)	0.58	17	1.45 (0.84–2.49)	0.18	1.55 (0.89–2.69)	0.12
≥4 cups/day	5	1.06 (0.43–2.60)	0.90	1.19 (0.48–2.95)	0.71	0	0.00 (—)	0.97	0.00 (—)	0.98
*P* for trend			0.71		0.53			0.52		0.37

For colorectal cancer incidence										
<1 cup/day	379	1.00		1.00		336	1.00		1.00	
1 cup/day	72	1.12 (0.87–1.44)	0.38	1.11 (0.86–1.43)	0.44	59	0.99 (0.75–1.30)	0.91	0.97 (0.73–1.28)	0.82
2–3 cups/day	88	1.19 (0.94–1.51)	0.14	1.21 (0.95–1.54)	0.12	44	1.05 (0.76–1.45)	0.77	1.04 (0.75–1.44)	0.81
≥4 cups/day	18	1.47 (0.91–2.37)	0.12	1.57 (0.97–2.55)	0.07	5	1.46 (0.60–3.56)	0.41	1.42 (0.57–3.50)	0.45
*P* for trend			0.04		0.03			0.55		0.61

HR, hazard ratio.

^a^Hazard ratio was adjusted for age, smoking, drinking, family history of colorectal cancer, education, body mass index, walking time, and regular meat consumption, and distict.

To control for the potential impact of subclinical symptoms of colorectal cancer at the baseline survey, we repeated the analysis by excluding cases occurring within two years after the baseline survey. Results after exclusion still showed a significant increase in the risk of colon cancer associated with increasing levels of coffee consumption among men (*P* for trend = 0.037): compared to men who consumed less than 1 cup of coffee per day, men who consumed 2–3 cups had an adjusted HR of 1.24 (95% CI 0.90–1.71), and those who consumed more than 4 cups had an adjusted HR of 1.82 (95% CI 1.00–3.32). On the other hand, coffee consumption in women was not associated with increased risk of colorectal cancer (data not shown).

Although we adjusted for lifestyle risk factors known to be associated with coffee consumption, such as smoking status, drinking status, BMI, meat consumption, and age, it remains possible that these factors could perturb the incident risk of colon and rectal cancer. To clarify the effect of these potential confounders, we performed additional analyses stratified by smoking status, drinking status, BMI, meat consumption, and age (Tables 3–7).

**Table 3.** Hazard ratio for colorectal cancer incidence for analysis of coffee consumption, stratified by sex and smoking status

Coffee consumption	Men						Women					
	
	Current			Never			Current			Never		
			
	Number of cases	HR^a^	*P* value	Number of cases	HR^a^	*P* value	Number of cases	HR^a^	*P* value	Number of cases	HR^a^	*P* value
For colon cancer incidence												
<1 cup/day	109	1.00		42	1.00		4	1.00		222	1.00	
1 cup/day	21	1.08	0.77	7	1.18	0.68	1	1.03	0.98	37	1.03	0.88
2–3 cups/day	34	1.21	0.36	10	1.85	0.09	5	4.71	0.04	17	1.46	0.23
≥4 cups/day	9	1.68	0.15	2	5.58	0.02	2	6.06	0.09	3	1.69	0.19
*P* for trend			0.13			0.01			0.02			0.60

For rectum cancer incidence												
<1 cup/day	59	1.00		29	1.00		2	1.00		70	1.00	
1 cup/day	14	1.36	0.31	9	2.13	0.05	0			10	0.84	0.62
2–3 cups/day	19	1.19	0.52	4	1.11	0.85	2	1.44	0.75	15	1.74	0.06
≥4 cups/day	2	0.67	0.59	2	6.31	0.01	0			0		
*P* for trend			0.91			0.12						0.20

For colorectal cancer incidence												
<1 cup/day	168	1.00		71	1.00		6	1.00		292	1.00	
1 cup/day	35	1.17	0.41	16	1.57	0.11	1	0.61	0.66	47	0.98	0.91
2–3 cups/day	53	1.21	0.26	14	1.52	0.16	7	3.65	0.04	32	1.00	0.99
≥4 cups/day	11	1.32	0.38	4	5.92	0.00	2	2.87	0.24	3	1.46	0.52
*P* for trend			0.20			0.01			0.04			0.80

HR, hazard ratio.

^a^Hazard ratio was adjusted for age, drinking, family history of colorectal cancer, education, body mass index, walking time, and regular meat consumption.

**Table 4.** Hazard ratio for colorectal cancer incidence for analysis of coffee consumption, stratified by sex and drinking status

Coffee consumption	Men						Women					
	
	Drinker			Non-drinker			Drinker			Non-drinker		
			
	Number of cases	HR^a^	*P* value	Number of cases	HR^a^	*P* value	Number of cases	HR^a^	*P* value	Number of cases	HR^a^	*P* value
For colon cancer incidence												
<1 cup/day	190	1.02		27	1.00		42	1.00		193	1.00	
1 cup/day	33	1.28	0.93	6	1.01	0.98	7	0.63	0.27	30	1.03	0.88
2–3 cups/day	44	1.74	0.16	10	1.21	0.63	7	0.77	0.53	16	1.46	0.23
≥4 cups/day	8	1.68	0.13	2	0.86	0.84	2	2.68	0.20	2	1.69	0.19
*P* for trend			0.06			0.85			0.98			0.78

For rectum cancer incidence												
<1 cup/day	107	1.00		17	1.00		9	1.00		63	1.00	
1 cup/day	25	1.43	0.11	3	0.80	0.72	2	0.83	0.82	9	0.86	0.69
2–3 cups/day	22	1.11	0.66	5	1.16	0.78	3	1.39	0.64	13	1.74	0.08
≥4 cups/day	3	1.08	0.89	2	2.37	0.28	0			0	0.00	0.99
*P* for trend			0.91			0.12			0.83			0.25

For colorectal cancer incidence												
<1 cup/day	297	1.00		44	1.00		51	1.00		256	1.00	
1 cup/day	58	1.16	0.30	9	0.95	0.89	9	0.66	0.26	39	0.96	0.83
2–3 cups/day	66	1.22	0.17	15	1.19	0.58	10	0.89	0.74	29	1.10	0.64
≥4 cups/day	11	1.49	0.20	4	1.33	0.61	2	1.99	0.37	2	1.04	0.95
*P* for trend			0.08			0.49			0.91			0.69

HR, hazard ratio.

^a^Hazard ratio was adjusted for age, smoking, family history of colorectal cancer, education, body mass index, walking time, and regular meat consumption.

**Table 5.** Hazard ratio for colorectal cancer incidence for analysis of coffee consumption, stratified by sex and BMI status

Coffee consumption	Men									Women								
	
	BMI < 18.5			18.5 ≤ BMI < 25.0			25.0 ≤ BMI			BMI < 18.5			18.5 ≤ BMI < 25.0			25.0 ≤ BMI		
					
	Number of cases	HR^a^	*P* value	Number of cases	HR^a^	*P* value	Number of cases	HR^a^	*P* value	Number of cases	HR^a^	*P* value	Number of cases	HR^a^	*P* value	Number of cases	HR^a^	*P* value
For colon cancer incidence																		
<1 cup/day	6	1.00		177	1.00		48	1.00		16	1.00		163			57	1.00	
1 cup/day	3	3.34	0.10	33	1.06	0.75	8	1.04	0.93	2	0.73	0.68	31	0.97	0.86	11	1.25	0.52
2–3 cups/day	6	5.06	0.01	38	1.14	0.48	10	1.05	0.89	2	0.89	0.88	14	0.64	0.12	8	1.35	0.45
≥4 cups/day	2	5.88	0.05	8	1.64	0.18	3	1.92	0.29	0			4	2.12	0.15	0		
*P* for trend			0.01			0.20			0.48			0.69			0.49			0.59

For rectum cancer incidence																		
<1 cup/day	6	1.00		99			25	1.00		5	1.00		52	1.00		16	1.00	
1 cup/day	1	1.49	0.72	23	1.43	0.13	2	0.42	0.25	1	1.42	0.77	11	0.82	1.08	1	0.49	0.50
2–3 cups/day	1	0.76	0.81	25	1.43	0.13	3	0.46	0.22	1	1.83	0.60	11	0.31	1.42	5	3.34	0.04
≥4 cups/day	0			5	1.94	0.16	0			0			0			0		
*P* for trend			0.74			0.06			0.11			0.62			0.61			0.08

For colorectal cancer incidence																		
<1 cup/day	12	1.00		276	1.00		73	1.00		21	1.00		215	1.00		73	1.00	
1 cup/day	4	2.40	0.14	56	1.19	0.24	10	0.82	0.56	3	0.80	0.72	42	0.99	0.97	12	1.11	0.74
2–3 cups/day	7	2.86	0.04	63	1.25	0.13	13	0.82	0.54	3	1.07	0.92	25	0.84	0.42	13	1.79	0.07
≥4 cups/day	2	3.64	0.12	13	1.74	0.06	3	1.13	0.84	0			4	1.51	0.42	0		
*P* for trend			0.03			0.03			0.73			0.87			0.75			0.16

BMI, body mass index; HR, hazard ratio.

^a^Hazard ratio was adjusted for age, smoking, drinking, family history of colorectal cancer, education, walking time, and regular meat consumption.

**Table 6.** Hazard ratio for colorectal cancer incidence for analysis of coffee consumption, stratified by sex and meat consumption status

Coffee consumption	Men									Women								
	
	Low meat consumption			Middle meat consumption			High meat consumption			Low meat consumption			Middle consumption			High meat consumption		
					
	Number of cases	HR^a^	*P* value	Number of cases	HR^a^	*P* value	Number of cases	HR^a^	*P* value	Number of cases	HR^a^	*P* value	Number of cases	HR^a^	*P* value	Number of cases	HR^a^	*P* value
For colon cancer incidence																		
<1 cup/day	75	1.00		69	1.00		35	1.00		70	1.00		67			50	1.00	
1 cup/day	14	1.12	0.71	12	0.77	0.41	12	1.64	0.15	10	1.01	0.97	16	1.00	0.99	11	0.99	0.98
2–3 cups/day	17	1.25	0.42	18	0.91	0.72	13	2.01	0.04	5	0.82	0.67	7	0.63	0.25	11	1.17	0.65
≥4 cups/day	2	1.03	0.97	5	1.53	0.37	4	2.98	0.05	3	7.19	0.00	1	1.76	0.58	1	0.74	0.77
*P* for trend			0.51			0.79			0.01			0.25			0.41			0.82

For rectum cancer incidence																		
<1 cup/day	46	1.00		40	1.00		24	1.00		29	1.00		17	1.00		12	1.00	
1 cup/day	6	0.79	0.60	10	1.221	0.58	8	1.90	0.12	6	1.239	0.64	5	1.119	0.83	2	0.71	0.66
2–3 cups/day	7	0.88	0.77	12	1.122	0.74	8	1.94	0.13	3	0.872	0.83	7	1.905	0.18	2	0.89	0.89
≥4 cups/day	3	2.51	0.14	1	0.599	0.62	1	1.25	0.83	0			0			0		
*P* for trend			0.54			0.99			0.22			0.68			0.31			0.61

For colorectal cancer incidence																		
<1 cup/day	121	1.00		109	1.00		59	1.00		99	1.00		84	1.00		62	1.00	
1 cup/day	20	1.00	0.99	22	0.93	0.77	20	1.78	0.03	16	1.08	0.78	21	1.02	0.92	13	0.96	0.89
2–3 cups/day	24	1.12	0.62	30	0.98	0.94	21	1.98	0.01	8	0.85	0.66	14	0.94	0.83	13	1.14	0.69
≥4 cups/day	5	1.59	0.32	6	1.21	0.65	5	2.36	0.08	3	3.89	0.03	1	1.12	0.91	1	0.58	0.60
*P* for trend			0.36			0.84			0.01			0.49			0.89			0.96

HR, hazard ratio.

^a^Hazard ratio was adjusted for age, smoking, drinking, family history of colorectal cancer, education, body mass index, and walking time.

**Table 7.** Hazard ratio for colorectal cancer incidence for analysis of coffee consumption, stratified by sex and age

Coffee consumption	Men						Women					
	
	40–59 years			60–79 years			40–59 years			60–79 years		
			
	Number of cases	HR^a^	*P* value	Number of cases	HR^a^	*P* value	Number of cases	HR^a^	*P* value	Number of cases	HR^a^	*P* value
For colon cancer incidence												
<1 cup/day	103	1.00		137	1.00		75	1.00		179	1.00	
1 cup/day	22	0.91	0.70	22	1.11	0.65	20	0.91	0.72	26	0.91	0.64
2–3 cups/day	31	0.86	0.46	27	1.52	0.05	14	0.63	0.12	13	0.81	0.48
≥4 cups/day	9	1.24	0.55	4	1.73	0.28	3	0.98	0.98	2	2.80	0.15
*P* for trend			0.89			0.03			0.21			0.84

For rectum cancer incidence												
<1 cup/day	52	1.00		87	1.00		30	1.00		52	1.00	
1 cup/day	18	1.57	0.10	10	0.82	0.55	7	0.93	0.87	6	0.74	0.49
2–3 cups/day	21	1.27	0.37	9	0.81	0.54	11	1.51	0.26	6	1.38	0.46
≥4 cups/day	4	1.30	0.62	1	0.73	0.75	0	0.00	0.99	0	0.00	0.99
*P* for trend			0.41			0.47			0.62			0.68

For colorectal cancer incidence												
<1 cup/day	155	1.00		224	1.00		105	1.00		231	1.00	
1 cup/day	40	1.13	0.50	32	1.00	1.00	27	0.92	0.69	32	0.87	0.47
2–3 cups/day	52	0.99	0.95	36	1.24	0.24	25	0.86	0.51	19	0.94	0.78
≥4 cups/day	13	1.27	0.42	5	1.36	0.50	3	0.76	0.64	2	2.25	0.26
*P* for trend			0.70			0.19			0.44			0.98

HR, hazard ratio.

^a^Hazard ratio was adjusted for smoking, drinking, family history of colorectal cancer, education, body mass index, walking time, and regular meat consumption.

Table 3 shows HRs for incident colorectal cancer associated with coffee consumption in subjects stratified by smoking status. Participants were classified dichotomously as current smokers or never smokers. Former smokers were excluded due to the small number of participants in this category. Table 4 shows HRs for incident colorectal cancer associated with coffee consumption in participants stratified by drinking status (current drinker or never drinker). We excluded former drinkers due to the small number of participants in this category. Table 5 shows HRs for incident colorectal cancer associated with coffee consumption in subjects stratified by BMI (<18.5 kg/m^2^, 18.5 kg/m^2^ to 25 kg/m^2^, or >25.0 kg/m^2^); Table 6 shows HRs by tertile of meat consumption; and Table 7 shows HRs by age. Consistent with the main findings in Table 2, these stratified analyses showed that coffee consumption was associated with an increased risk of colon cancer among men.

## DISCUSSION

In our analysis of a large population-based prospective study, we found that coffee consumption increased the risk of colon cancer among Japanese men. In contrast to these results, coffee consumption by women was not associated with incident risk of colon cancer. We used the data from a large-scale cohort study in Japan. Data for the analysis of coffee drinking frequency at baseline included 687 cases of colon cancer and 317 cases of rectal cancer.

The reason for the different results between sexes is unclear but might be at least partially attributable to residual confounding effects in men. Most subjects who consumed a large amount of coffee were smokers, and the proportion of smokers among men was higher than that among women (Table 1). Although we adjusted for lifestyle risk factors, such as smoking, drinking, and meat consumption, the effects of these lifestyle risk factors might not have been completely removed due to their strong associations with coffee consumption. One way to account for the influence of these factors is to analyze the groups after stratification by smoking status, BMI, meat consumption, and drinking status (Tables 3–7). Consistent with the findings from the primary analysis (Table 2), stratified results showed that coffee consumption was associated with an increased risk of colon cancer among men.

Although many studies in various populations have examined the association between coffee consumption and colorectal cancer, epidemiologic evidence for an effect has been inconsistent. In fact, a 2007 report by the World Cancer Research Fund and the American Institute for Cancer Research determined that no firm conclusions on this association could be reached because of inconsistent epidemiologic evidence. Several case-control studies have reported null associations between coffee consumption and colorectal cancer risk.^19^^–^^23^ In contrast, other case-control studies reported a modest but statistically significant decrease in colon cancer risk,^24^^–^^26^ whereas one study reported a statistically significant increase in risk among men.^27^ Epidemiologic evidence from cohort studies has been inconsistent.^3^^–^^13^ While most previous cohort studies were conducted in Western countries, several cohort studies in Asia have been conducted, including several major cohort studies on the risk of colorectal cancer from Japan.^11^^–^^13^ However, results from these studies were also inconsistent. The JPHC Cohort Study and the Takayama Cohort Study suggested that coffee consumption may lower the risk of colon cancer in women,^11^^,^^13^ whereas the Miyagi Cohort Study concluded that consumption is not associated with risk of colorectal cancer in either sex.^12^ Elsewhere in Asia, the Singapore Chinese Health Study found a null association between coffee intake and risk of colorectal cancer overall but also found that consumption may protect against smoking-related advanced colon cancer.^28^

A few recent meta-analyses of prospective studies have shown rather wide discrepancies in findings. Je et al confirmed that coffee drinking is not associated with colorectal cancer risk.^14^ On the contrary, Yu et al analyzed results from 15 worldwide cohorts and reported that consumption had a significant inverse association with colorectal cancer risk.^29^ Further, Li et al reported that coffee consumption significantly decreased the risks of colorectal cancer and colon cancer, especially in Europe and for females.^15^ In a pooled analysis of prospective cohort studies, Zhang et al found no association between coffee consumption and colon cancer.^30^ Contrary to our expectation, our results showed that coffee consumption increased the risk of colon cancer among men. This observation may be due to the complex biological effects of coffee.

The biological mechanisms of coffee’s effects have been examined in many studies.^31^^–^^33^ Roasted coffee is a complex mixture of more than a thousand chemicals. Coffee intake may increase colonic motility, thereby decreasing the exposure of epithelial cells to potential carcinogens in the colon.^34^ Also, coffee consumption may reduce the synthesis and secretion of bile acids, which are known to be potential promoters of carcinogenesis.^35^ Thus, these complex compounds in coffee with their various effects may explain the lack of association or the inconsistent results observed to date.

Our study has several limitations. First, data were collected at the baseline survey only, and the consumption of coffee was assessed by self-report. Thus, some measurement error at baseline was inevitable. Second, we did not collect details of coffee consumption, such as the use of caffeinated or decaffeinated coffee, and the method of coffee preparation (eg filtered or boiled). Although coffee preparation and consumption habits may change considerably over time and vary seasonally and geographically, our present and previous published studies lack these considerations. These limitations may have biased or produced inconsistencies in the epidemiologic evidence for the effect of coffee on the incidence of colorectal cancer, and further detailed cohort studies which take account of these points are required.

In conclusion, a large-scale population-based cohort study showed that coffee consumption increases the risk of colon cancer among Japanese men. In contrast to these results, coffee consumption by women is not associated with the incident risk of colorectal cancer.

## ONLINE ONLY MATERIAL

Abstract in Japanese.
