# Supplementary material for: Coffee Consumption and Risk of Colorectal Cancer: The Japan Collaborative Cohort Study
Source: J Epidemiol. 2014 Sep 5;24(5):370–8. doi: 10.2188/jea.JE20130168 (PMC4150007; doi:10.2188/jea.JE20130168)
Supplement: Abstract in Japanese. [file je-24-370-s001.pdf]

コーヒー摂取と大腸がんリスク：JACC Study

山田宏哉[1]、川戸美由紀[1]、青山典裕[1]、橋本修二[1]、鈴木康司[2]、若井建志[3]、鈴木貞夫[4]、渡邊能行[5]、玉腰暁子[6]、JACC Study Group
[1]藤田保健衛生大学医学部衛生学講座、[2]藤田保健衛生大学医療科学部公衆衛生学、[3]名古屋大学大学院医学研究科予防医学、[4]名古屋市立大学大学院医学研究科公衆衛生学分野、[5]京都府立医科大学大学院医学研究科地域保健医療疫学、[6]北海道大学大学院医学研究科公衆衛生学分野

【背景】
疫学研究によりコーヒー摂取と様々な健康状態との関連が報告されている。今回、コーヒー摂取と大腸がんとの関連を前向きコホート研究により評価することを目的とする。

【方法】
JACC Study における 1988 年から 2009 年まで追跡した 58,221 人（男性 23,607 人、　女性 34,614 人）のデータを用いた。ベースライン後の 738,669 人年の追跡により、結腸がん罹患 687 人（男性 355 人、女性 332 人)、直腸がん 314 人（男性男性 202 人、女性 112 人）が観察されている。大腸がん罹患の有無に基づいて、ハザード比と 95%信頼区間を、コックス回帰により交絡変数を調整して推定した。
【結果】
結腸がんについて、コーヒー摂取が 1 日 1 杯以下の男性に比べて、1 日 2～3 杯のコーヒー摂取をする男性で HR が 1.26 (95%信頼区間 0.93-1.70)、1 日 4 杯以上の男性で HR が 1.79 (95%信頼区間 1.01-3.18)を示した。一方、女性では有意な関連は認められなかった。また、男女とも直腸がんでは関連が認められなかった。
【結論】
JACC study の解析結果では、男性においてコーヒー摂取は結腸がんリスクの上昇を示した。

キーワード：コーヒー、大腸がん、罹患、前向き研究、the Japan Collaborative Cohort Study
